# Testing international techniques for the radiographic assessment of third molar maturation

**DOI:** 10.4317/jced.58916

**Published:** 2021-12-01

**Authors:** Vanessa Sartori, Ademir Franco, Maria-Salete Linden, Moisés Cardoso, Dora de Castro, Angélica Sartori, Cauane Silva, Micheline Trentin, João-Paulo De Carli

**Affiliations:** 1 MSc. University of Passo Fundo, School of Dentistry, Postgraduate Program in Dentistry, Passo Fundo, Rio Grande do Sul, BrazilMSc. University of Passo Fundo, School of Dentistry, Postgraduate Program in Dentistry, Passo Fundo, Rio Grande do Sul, Brazil; 2 PhD. São Leopoldo Mandic School of Dentistry, Campinas, São Paulo, Brazil; Department of Therapeutic Dentistry, Institute of Dentistry, Sechenov University, Russia; Forensic Dentistry, Centre of Forensic and Legal Medicine and Dentistry, University of Dundee, Scotland/UK; 3 PhD. University of Passo Fundo, School of Dentistry, Postgraduate Program in Dentistry, Passo Fundo, Rio Grande do Sul, Brazil; 4 DDS. University of Passo Fundo, School of Dentistry, Postgraduate Program in Dentistry, Passo Fundo, Rio Grande do Sul, Brazil; 5 Ind. Sol Private law firm, Passo Fundo, Rio Grande do Sul, Brazil; 6 PhD. International Center for Equity in Health, Federal University of Pelotas, Pelotas, Rio Grande do Sul, Brazil

## Abstract

**Background:**

To assess the radiographic third molar maturation with internationally developed techniques for age estimation.

**Material and Methods:**

The study analyzed 1,062 panoramic radiographs of patients treated at the School of Dentistry of the University of Passo Fundo/RS/Brazil, between 2009 and 2020. The patients were separated into ages between 15.00 and 23.99 years and, for each radiograph, the third molars were classified into stages from 1 to 10, with the subsequent application of one of the formulae proposed by Gunst et al. A dichotomous variable indicating whether each individual was younger or older than 18 was calculated from the chronological age of the individuals. A logistic regression model was adjusted to assess whether the third molar stages are correlated with the age of individuals.

**Results:**

The error indicators between estimated and chronological ages showed that mean errors closer to zero are seen in the 18-18.9 and 17-17.9 age groups, respectively. Male individuals were earlier in terms of dental mineralization but there were no significant differences between sexes regarding the applicability of the method. The ROC curve shows that the analysis of a single third molar for age estimation gives a maximum of 70.4% reliability.

**Conclusions:**

The moderate performance of the technique tested in the present study justify future country-specific corrections to improve age estimation from the radiographic assessment of third molar maturity.

** Key words:**Dental age estimation, forensic dentistry, radiology, third molar.

## Introduction

Dental age estimation becomes increasingly challenging with the progressive development of permanent teeth (1). In subadults, forensic odontologists have the last chance to benefit from tooth development to support dental age estimation practices (2). Third molars are the only developing teeth that might be used to answer legal questions on age thresholds of judicial interest, especially regarding the legal majority (3,4). Applications of third molar age estimation are mainly related to the living, especially in cases of asylum seekers (5,6), criminal imputability (7), and sports competitions (8). Applications to the deceased involve identifying cadavers and skeletal remains in single cases (9) or mass disasters (10).

Third molar visualization for dental age estimation was previously described with static imaging such as panoramic radiographs (11), and dynamically with computed tomography navigation (12) and magnetic resonance imaging (13). In 2003, Gunst *et al*. (14) proposed multiple regression formulae for third molar age estimation from an original sample of 2,513 panoramic radiographs of Belgian subadults aged between 15.7 and 23.3 years. The formulae showed the known chronological age of patients as independent variables and third molar developmental stages as dependent variables (14). The third molar stages used were proposed by Köhler *et al*. (15) in 1994, after an adaptation of the original system proposed by Gleiser & Hunt (16) in 1955.

So far, the formulae proposed in the study by Gunst *et al*. (14) have never been tested in Brazilians. The importance of having population-specific testing might reflect forensic applications to Brazilians entering the international territory undocumented, or even in dental age estimation practice in Brazil. Case reports already highlighted the importance of country-specific studies on dental age estimation methods, especially when it comes to dental age estimation practice to assess the age of legal majority (17). In these cases, third molar development may be analyzed to help on elucidating whether or not an individual is above or below certain age thresholds of legal interest (17). Testing the international formulae in a Brazilian population is, therefore, justified to populate the forensic armamentarium with scientific tools. This scenario depicts the importance of testing international methods in the country toward optimal forensic practice.

Based on the exposed, the present study aimed to apply, for the first time, the formulae proposed by Gunst *et al*. (14) for third molar age estimation in a sample of panoramic radiographs of a Brazilian population, analyzing the hypothesis that such a method will be useful for the population studied, regardless of their ancestry.

## Material and Methods

This observational cross-sectional study was approved by the Human Research Ethics Committee of the University of Passo Fundo/RS/Brazil (protocol #3.688.526).

The sample consisted of 1,062 panoramic radiographs from Brazilian women (n = 623; 58.66%) and men (n = 439; 41.34%). The mean age of the sample was 19.7 ± 2.4 years for women and 20.2 ± 2.3 years for men (Table 1). The sample was retrospectively collected from an existing database of panoramic radiographs from 9,653 patients subjected to dental treatment at a Dentistry School in southern Brazil, between 2009 and 2020. The images were acquired with an Eagle device (Dabi Atlante, Ribeirão Preto, SP, Brazil) set with 75 kVp and 8 mA. From the clinical records of each patient, sex, date of birth, and date of image acquisition were registered.

**Table 1:**
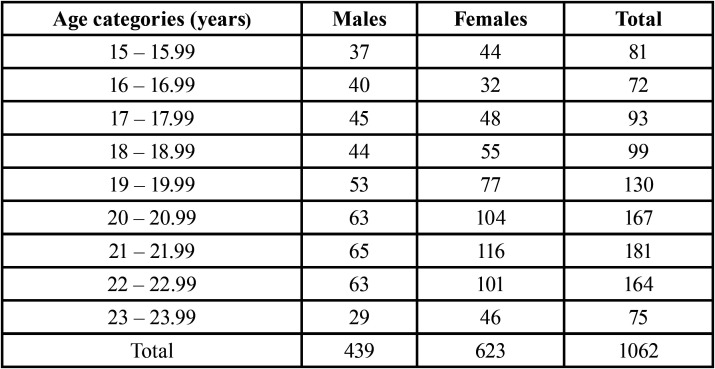
Sample distribution (n) based on sex and age.

The inclusion criteria consisted of Brazilian women and men aged between 15 and 23.99 years with a panoramic radiograph taken for dental treatment purposes showing at least one third molar. The exclusion criteria consisted of images with clear evidence of trauma in the mandible and maxilla, bone lesions and signs of skeletal deformity, low-quality radiographs, and patients showing all existing third molars with decay, fracture, restoration/obturation, and transversal position.

The images were analyzed according to the staging technique by Gleiser & Hunt (16), modified by Köhler *et al*. (15). This technique requires classifying third molar crown-root development into 10 ordinal stages. The three initial stages are established to classify crown formation, namely, stage 1: half of the crown developed (Cr ½); stage 2: three-quarters of the crown developed (Cr ¾); and stage 3: crown completely developed (Crc). The five following stages describe root development, more specifically, stage 4: initial root development (Ri); stage 5: a quarter of the root developed (R ¼); stage 6: half of the root developed (R ½); stage 7: three-quarters of the root developed (R ¾); and stage 8: root completely developed (Rc). The two final stages represent late third molar development and indicate half of the apex developed (Stage 9: A ½) and apex completely developed (Ac). As third molars usually appear as multi-rooted teeth, the staging process always considered the least developed root. All third molars present in the panoramic radiograph were classified.

Before the classification process, the main observer was trained by a supervisor experienced in dental age estimation. Next, a sample of 100 panoramic radiographs (randomly selected from the main sample) was used to calculate intra- and inter-observer reproducibility. For intra-observer reproducibility, an interval of 30 days was established between initial (n = 100) and main (n = 1.062) radiographic analyses. Weighted kappa values indicated excellent intra- and inter-observer reproducibility values that reached 0.84 and 0.85, respectively.

The variables considered in the present study were patient sex, documental age (deducted between the date of image acquisition and date of birth), third molar stage, and estimated dental age. The estimated age was calculated by quantifying the third molar allocated stages into scores from 1 to 10. Next, the scores obtained replaced the “x” variables in the first-degree equations proposed by Gunst *et al*. (14). The equations are sex-dependent and were calculated based on an original Belgian reference of juveniles. To date, this is the first application of the Belgian equations for age estimation in a Brazilian population.

The data were analyzed with descriptive statistics of absolute (n) and relative (%) frequencies of the number of panoramic radiographs, male and female individuals, and the number of third molars per type. The difference between estimated and chronological ages was calculated and presented as central tendency and dispersion (mean error, mean absolute error, and root mean squared error). The age of 18 was set as a threshold (dichotomous variable) to classify individuals as minors (<18) or not (≥18). Similar to Gunst’s (14) original study, the probability rates of an individual being 18 or older in the presence of completely developed third molars were calculated. The number of minors with the third molar in stage H was registered. Receiver Operating Characteristic (ROC) curves were calculated for each third molar to assess and illustrate the predictive power of teeth to distinguish minors and adults. Statistical analyses were performed with the Stata™ software 16.1 (StataCorp LLC, College Station, TX, USA).

## Results

Table 2 shows the mean estimated and chronological ages for each age interval of one year between 15 and 23.99 years, and their respective standard deviations, for men and women. The mean error between estimated and chronological ages was lower in the age intervals from 17 to 18.99 years, for men and women. In the age interval from 17 to 17.99 years, the mean difference between estimated and chronological ages was 0.4 (standard deviation = 1.3), while in the interval between 18 and 18.9 years, the mean error was zero (standard deviation = 1.2). The worst outcomes were observed in age intervals in the upper bound of the sample, namely in the age interval between 21 and 23.99 years, with overestimates from -2.1 to -4.5 years. Table 3 revealed that individuals with multiple third molars in stage 10 (completely developed) were more likely to be 18 or older. The probability was directly proportional to the increase in developed third molars. In male individuals, a single third molar in stage 10 (upper right third molar; #18) led to a probability of 73.4% of being an adult, while in female individuals, the same tooth led to the increased rate of 84.4%.

**Table 2:**
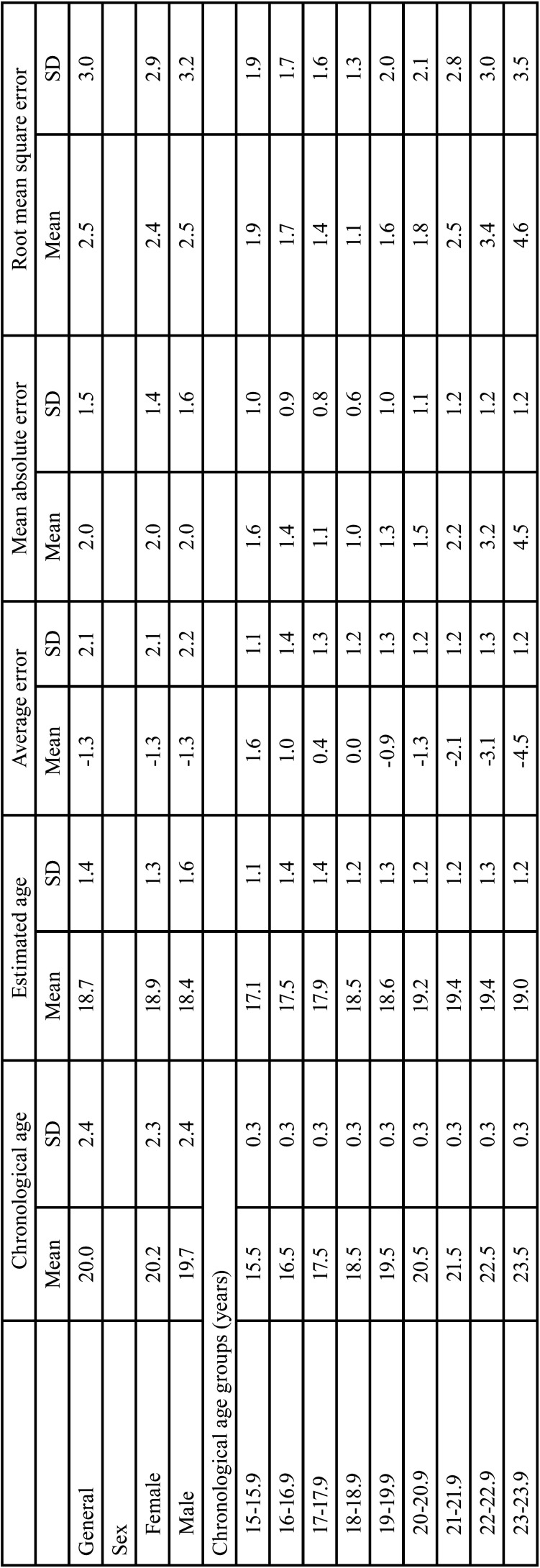
Chronological and estimated ages according to sex and age group. Error indicators show differences between the estimated age deducted from the chronological age.

**Table 3:**
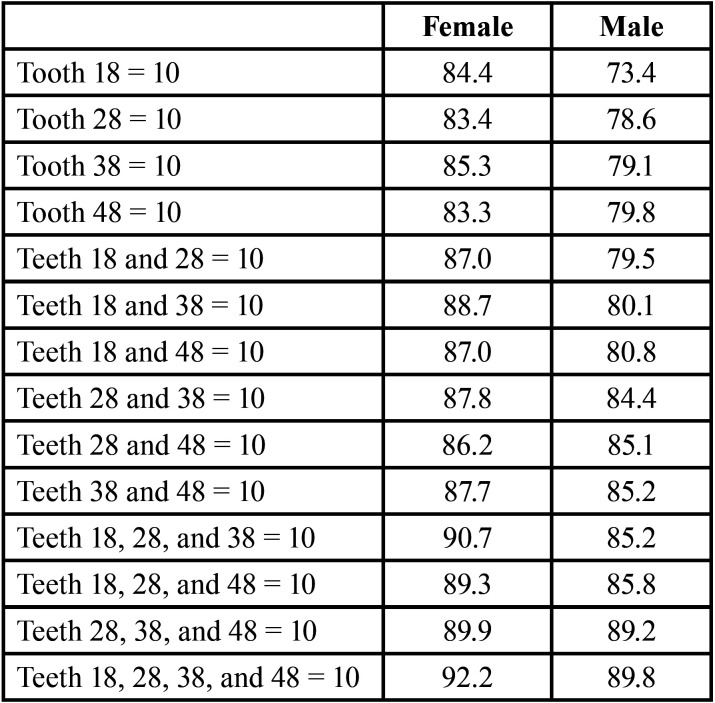
Probability of an individual, according to sex, to be 18 years old or more in case of complete development of third molars.

**Figure 1:**
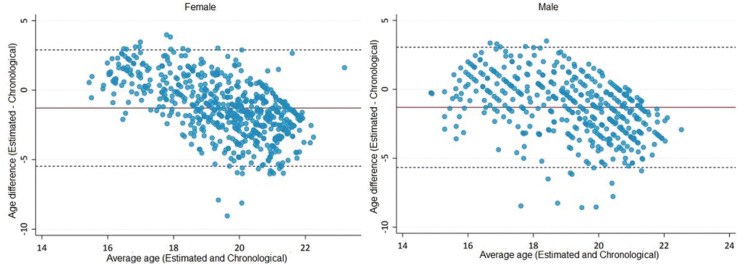
Bland-and-Altman graph for analysis of agreement between estimated and chronological ages for females (left) and males (right). Dashed lines indicate the limits of agreement and solid lines indicate the mean difference between ages.

Figure 1 illustrates the higher dispersion of the difference (error measure) between estimated and chronological ages in female individuals. The differences between mean errors were, however, close to zero and not statistically significant between male and female individuals (*p*>0.05).

**Figure 2:**
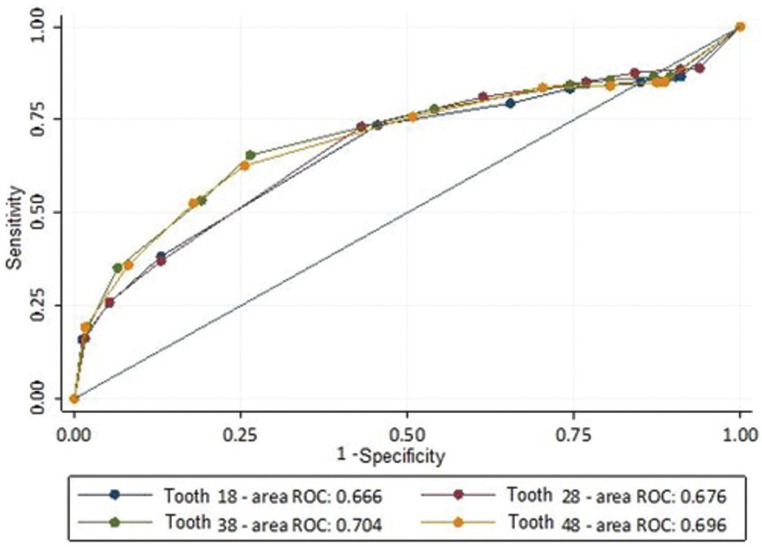
ROC curve for each tooth indicating potential cut-off points for identifying individuals aged ≥18 years.

The ROC curves in Figure 2 show mandibular third molars with the highest area under the curve (AUC). The accuracy expressed by the AUC, however, was ≤ 0.70 for each tooth, which indicates a poor diagnostic power of the tooth stages within the international equations for dental age estimation of Brazilian individuals.

## Discussion

Dental age estimation outcomes may emerge in different forms depending on the techniques applied in practice. Specific approaches lead to a dichotomous answer, namely minor or not (18). Other approaches lead to continuous variables and an estimated age is obtained as a result (14,19-21). The descriptive outcomes of the present study show that the international equations originally proposed by Gunst *et al*. (14) in a Belgian population can deliver age estimates with accepTable mean errors for Brazilian juveniles, especially considering the outcomes that do not include the lower and upper bounds of the sampled age interval. This means that the method was effective to estimate the dental age of juveniles younger than 21 years, in which the mean error was below 1.6. The increasing error rates following sample aging may be justified by the age-related parameter of choice (i.e. dental development) that progresses to an end toward the age of 21 (22). Inferences on the age of majority, however, are the type of outcome not supported by the present study after applying the Belgian equations in a Brazilian population. To sustain this argument, ROC curves with poor outcomes showed that third molars were not predictive enough to properly distinguish minors and adults (outcomes ≤ 0.704). From a legal perspective, applying the proposed approach to have a dichotomous outcome around the age of 18 would be a dangerous procedure, considering the possibility of error. In particular, ROC curves with such low specificity values could lead to an increased number of false positives (minors classified as adults), which should be avoided with utmost importance in the forensic field, especially because of the negative impact of its consequences on individual human rights.

The origin of the sample is usually considered one of the justifications behind the negative outcomes of validation studies on foreign samples. In this study, the Belgian equations were tested in a Brazilian sample but despite the distant geographic location between populations, the Brazilian sample was collected from the southernmost state, which is a region with strong (Caucasian) European ancestry, especially from Germany and Italy.

The European influence in the Brazilian sample is part of the broad spectrum of heterogeneity (23) in the country and could be responsible for enclosing dental development between countries. Willershausen *et al*. (24) corroborate the developmental similarity between individuals with different ancestries (24). More specifically for human third molars, Thevissen *et al*. (25) suggested a similar development across nine countries – including Brazil and Belgium. The authors used the same third molar classification system of the present study, making the results even more comparable (25). It must be noted that this is the first time the Belgian formulae were applied and tested in a Brazilian population. A similar study was performed in an Indian population with highly similar objectives. Contrasting with the present study, the authors sampled 268 radiographs of individuals aged between 14 and 23 years. Using a distinct statistical analysis, the authors found a correct allocation rate of 78% of minors. Their conclusion highlighted the applicability of the method to estimate the age of Indians but also emphasized the restricted applicability of the formula to distinguish minors and adults (26). In this context, the present study converges to support the applicability of the method when estimating the age is a requirement. However, if the application is requested to aid legal decisions based on the age majority (18, in this case), such as asylum seekers, criminal imputability, or sports players, careful steps must be taken. Specific methods designed to allocate individuals within dichotomous outcomes should be used instead.

A parallel outcome of the present study was the analysis of probabilities for the allocation of individuals based on the position and number of third molars in stage 10 (Ac). According to the present findings, there was a probability of 89.8% of male individuals being adults when all third molars were in stage 10. In female individuals, the probability increased to 92.2% in the same conditions. Similarly, high values (96.3% and 95.1%, respectively) were detected by Gunst *et al*. (14) in the original study reporting the equations. Moreover, with the same classification system, Bagherpour *et al*. (27) found probability rates of 95.6% among men and 100% among women (27). These outcomes not only confirm the important role of combined Ac evidence for investigating the age majority but also corroborate the concept of similar third molar development among different populations.

From the inherent nature of the referenced studies (14), the upper bound of the samples was set around the age of 23, restricting but not censoring stage 10. Future studies with censoring strategies are recommended to further understand the role of late third molar development in the allocation of minors and adults. Understanding more clearly the upper and lower boundaries of stage 9, for instance, could be an additional tool to be explored regarding the calculation of probabilities. Table 3 has an illustrative value to the present study and must not be taken as a shortcut to forensic dental age estimation in practice. Again, the Belgian equations can be used for age estimation in the Brazilian population but are limited for dichotomous outcomes around the age of majority.

The continental territory of Brazil hinders a country-size validation of the Belgian equations. Instead, the present study indicates that the equations were applicable and valid to estimate dental age in the southernmost state of Brazil. Future studies with different samples from other geographic locations are encouraged. The Belgian equations for dental age estimation from third molar development are useful and applicable to Brazilians from the southernmost state. The allocation of individuals as minors or adults, however, is not recommended based solely on this method.
